# An actor-critic model of saccade adaptation

**DOI:** 10.1186/1471-2202-14-S1-P425

**Published:** 2013-07-08

**Authors:** Manabu Inaba, Tadashi Yamazaki

**Affiliations:** 1 Graduate School of Informatics and Engineering, The University of Electro-Communications, 1-5-1 Chofugaoka, Chofu, Tokyo 182-8585, Japan

## 

The basal ganglia and the cerebellum are subcortical structures indispensable for voluntary motor control and motor learning. They are thought to perform reinforcement learning and supervised learning, respectively, and interact with each other [1]. Yet, how these structures and their learning mechanisms interact remains unknown.

**Figure 1:**
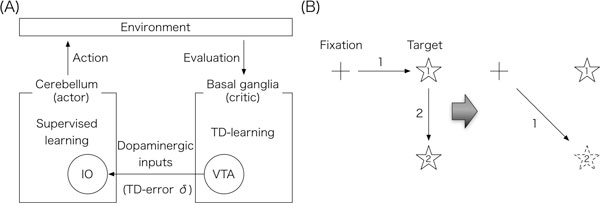
**(A) Proposed scheme of interaction between the basal ganglia and the cerebellum as an actor-critic model**. (B) Illustration of direction-adaptation of saccades.

In this study, we propose a model of interaction between the basal ganglia and the cerebellum for voluntary motor control and motor learning. We consider that the basal ganglia performs temporal difference (TD) learning. Specifically, according to the electrophysiological experiments [2], we assume that neurons in ventral tegmental area (VTA), a part of the basal ganglia, represent the value of delta, the prediction error of TD-learning. On the other hand, we consider that the cerebellum generates motor commands through supervised learning, for which the inferior olive (IO) provides teacher signals. Here, based on the anatomical findings of dopaminergic inputs from VTA to the IO [3], we assume that the cerebellum can receive the information of TD-prediction error as teacher signals via the IO. In the end, we propose a scheme of the interaction between the basal ganglia and the cerebellum as an actor-critic model in reinforcement learning (Figure 1A, [4]).

We adopt the proposed scheme to double-step adaptation of saccade, which is voluntary eye movement and is mediated by a distributed network including both the basal ganglia and the cerebellum. A double-step saccade adaptation paradigm called direction adaptation goes as follows (Figure 1B). Initially, the eye is fixated at the center position. Next, a target appears at a certain position, and the eye moves to the target (first saccade). When the saccade starts, the target is immediately removed and reappears to another position. In turn, the eye moves to the second target (corrective saccade). By repeating many trials, when the first target appears, the eye moves to the position of the expected second target. Our proposed model reproduces this direction-adaptation of saccades. These results suggest that the interaction between the basal ganglia and the cerebellum as an actor-critic model provides a powerful motor control and learning mechanism.
